# GABA_A_ receptor agonists modulate intracellular Ca^2+^ levels in activated human CD4^+^ T cells

**DOI:** 10.3389/fimmu.2026.1693150

**Published:** 2026-05-08

**Authors:** Sergiy V. Korol, Zhe Jin, Amol K. Bhandage, Hayma Hammoud, Bryndis Birnir

**Affiliations:** Department of Medical Cell Biology, Uppsala University, Uppsala, Sweden

**Keywords:** Ca^2+^ response, GABA, ion channels, TACA, ρ subunit

## Abstract

GABA binding to GABA_A_ receptors induces transient elevation in intracellular Ca^2+^ concentrations in activated human CD4^+^ T cells. Here we examined the Ca^2+^ response evoked by the GABA_A_ receptor agonists THIP, muscimol, isoguvacine, and TACA and further, effects of K_V_1.3 and KCa3.1 channel blockers on the GABA-evoked Ca^2+^ signal. The cells were activated by anti-CD3 antibody and cultured for 72 h before the effects of agonists and antagonists on intracellular Ca^2+^ levels were examined by Ca^2+^ imaging. mRNA sequencing, qPCR, and immunofluorescence imaging identified the GABA_A_ subunits expressed. Both the half-maximal concentration for activation (the apparent affinity (EC_50_)) and the evoked, peak cellular Ca^2+^ response varied among the agonists. The agonists EC_50_ values varied more than 1,000-fold, but the compounds can, nevertheless, all be classified as high-affinity agonists (EC_50_ < 1 μM). The EC_50_ sequence was as follows: GABA (0.005 nM) ≈ THIP (0.005 nM) < muscimol (0.162 nM) < TACA (2.2 nM) < isoguvacine (54.4 nM). In contrast, the peak increase in the cellular Ca^2+^ signal followed the sequence: GABA >> THIP > muscimol > isoguvacine > TACA. For all agonists, the transient Ca^2+^ signal was attenuated at micromolar concentrations. ShK-Dap22 and TRAM-34 that block K_V_1.3 and KCa3.1 channels, respectively, inhibited the GABA-activated Ca^2+^ response. The CRAC channel inhibitor YM58483 reduced the Ca^2+^ current by 40%. The pharmacology is consistent with the ρ2 GABA_A_ receptor subunit being a part of the high-affinity GABA_A_ receptors expressed in human CD4^+^ T cells. The agonists at the ρ2-containing GABA_A_ receptors induced intracellular Ca^2+^ signals in human CD4^+^ T cells that were related to the agonist concentration. All tested agonists have a high apparent affinity but differ in efficacy at the receptors. Ca^2+^ signaling enables high-throughput assays for fast screening of potential drugs aimed at GABA_A_ receptors regulating the T cell functions.

## Introduction

γ-Aminobutyric acid is a well-known inhibitory neurotransmitter and activates GABA_A_ and GABA_B_ receptors in the central nervous system (CNS) and the periphery (1–3). The highest concentration of GABA is in the brain (4), but physiologically meaningful levels of GABA are normally present in the blood (5–7) and various organs, including the pancreatic islets, lymph nodes, testes, and pituitary glands (2, 4). Immune cells do express a variety of receptors activated by their respective neurotransmitters (8). In the plasma membrane of T cells, high-apparent-affinity (EC_50_ < 1 μM) GABA_A_ receptors are present (9–11). Recently, in human CD4^+^ T cells, we recorded currents with a pharmacological profile resembling ρ-containing GABA_A_ receptors (12). In studies using expression systems, the pharmacology of homomeric ρ GABA_A_ receptors is significantly different from the more common heteromeric αβγ or αβδ GABA_A_ receptors (1, 13).

We have shown in human CD4^+^ T cells that the activation of GABA_A_ receptors by GABA leads to a graded, transient increase in intracellular Ca^2+^ signal (12). Interestingly, the GABA EC_50_ values of the Ca^2+^ signal and values for the GABA-activated GABA_A_ receptor currents differed by a factor of approximately 10 (12). Here we examined if the GABA_A_ receptor agonists—muscimol, TACA, isoguvacine, and THIP—similarly induce elevations in intracellular Ca^2+^ concentrations in the activated CD4^+^ T cells. We further examined whether the GABA-activated Ca^2+^ signal was affected by the activation of potassium channels. The pharmacological profile and expression of subunits confirm the presence of high-affinity ρ2-containing GABA_A_ receptors in the cells that effectively induce Ca^2+^ transients when activated by the GABA_A_ receptor agonists.

## Materials and methods

### Human CD4^+^ T lymphocyte isolation and activation

Human blood samples and blood buffy coats were obtained from Uppsala University Hospital (Uppsala Akademiska sjukhuset), the protocol of which was approved by the Regional Research Ethics Committee in Uppsala (Dnr 2013/347). All individuals who donated blood were voluntarily recruited. Peripheral blood mononuclear cells (PBMCs) were isolated from blood samples using density-gradient centrifugation with Ficoll-Paque™ PLUS (Sigma Aldrich, Sweden). Subsequently, CD4^+^ T lymphocytes were isolated from the PBMCs by negative selection, utilizing a human CD4^+^ T lymphocyte isolation kit (Miltenyi Biotec, Germany) as per the manufacturer’s instructions. The freshly isolated CD4^+^ T lymphocytes (1 million cells/mL) were suspended in human plasma-like medium (HPLM) (Gibco, Fisher Scientific, Sweden) containing 10% heat-inactivated dialyzed fetal bovine serum (Gibco, Fisher Scientific, Sweden), 100 μg/mL streptomycin, 100 U/mL penicillin (Gibco, Fisher Scientific, Sweden), and 5 μM β-mercaptoethanol (Gibco, Fisher Scientific, Sweden). The cells were activated in a 96-well plate (1 × 10^5^–1.2 × 10^5^ cells/well) with 3 μg/mL plate-bound anti-CD3 antibody (BD Biosciences, USA) for 72 h. Insulin (1 nM) was added at 48 h after the activation of the cells. The anti-CD3 concentration (5 μg/mL) used by Jacobs et al. (14) is similar to the one employed in this study. They showed that when anti-CD3 alone is used, the proliferation of the T cells was approximately one-third of the maximal stimulation obtained when co-stimulated with anti-CD28 at the maximal concentration of anti-CD3 (5 μg/mL). Akt-dependent signaling is activated by insulin at 48 h post-activation and is reminiscent of the anti-CD28-evoked signaling (14).

### Calcium imaging

Calcium imaging was performed as previously described (12). In brief, cells were loaded with 3 μM Fluo-8 AM calcium indicator (AAT Bioquest, Pleasanton, CA, USA) for 10 min at 37 °C and then seeded onto coverslips coated with 5% (3-aminopropyl)triethoxysilane (Sigma Aldrich) for 20 min at 37 °C. Calcium imaging was performed using a Zeiss LSM700 confocal microscope with a ×20 objective, capturing images at 0.5-s intervals. The recording solution was prepared by mixing two types of RPMI 1640 media, one containing 11 mM glucose (Gibco, Fisher Scientific, Sweden) and the other is glucose-free (Gibco, Fisher Scientific, Sweden). They were mixed in a 1:1 ratio, giving a final glucose concentration of 5.5 mM. The drugs were diluted in the recording solution and perfused into the chamber at specific concentrations using a peristaltic pump. After each drug application, the cells were perfused with the recording solution only to remove the drug. When the cells were again in the recording solution only, then the next test drug concentration was perfused into the chamber. This was done so that the receptors/cells had always been in a drug-free solution before a drug application, thus avoiding potential cumulative drug effects such as run-down or desensitization. The area around the individual cells was marked, and absolute fluorescence intensity values (F) were extracted using ZENBlue software. The relative intensity (*F*/*F*_median_) for each cell was calculated for every recorded image and plotted against time. The peak relative intensity during the drug application was considered the drug’s response. Data are presented for all cells as scatter plots and box and whisker plots.

### Electrophysiological patch-clamp recordings

To study the effect of YM58483 and picrotoxin (PTX) on CRAC-channel-mediated currents, the electrophysiological patch-clamp recordings were done in the whole-cell configuration. The extracellular solution was composed of the following (in mM): 155 NaCl, 4.5 KCl, 2 CaCl_2_, 1 MgCl_2_, 5 HEPES, and 5.6 glucose (pH 7.4, adjusted with NaOH). The pipette solution consisted of the following (in mM): 140 Cs methanesulfonate, 10 NaCl, 3 MgCl_2_, 10 HEPES, and 10 EGTA (pH 7.3, with CsOH). We further examined the recovery of currents during repeated drug applications to evaluate the level of run-down and desensitization, if present. In these experiments, in order to keep the intracellular chloride concentration intact, gramicidin (2.6 μg/mL) was used to obtain the perforated configuration. The extracellular solution (in mM) contained the following: 157 NaCl, 4.5 KCl, 0.5 CaCl_2_, 1 MgCl_2_, 5 HEPES, and 5.6 glucose (pH 7.4, adjusted with NaOH), and the pipette solution consisted of the following (mM): 149 KCl, 2 CaCl_2_, 1 MgCl_2_, 1 NaCl, 10 HEPES, and 5.6 glucose (pH 7.3, adjusted with KOH). The patch-clamp pipettes were produced from borosilicate glass and had a resistance range of 8–11 MΩ when filled with the pipette solution and brought into contact with the extracellular solution. Recordings were performed with the help of an Axopatch 200B amplifier, filtered at 2 kHz, and digitized at 10 kHz with an analog-to-digital converter. For the recordings, the Clampex 10.5 (Molecular Devices, San Jose, CA, USA) program was used.

For current recordings, the following procedure was used: 200-ms voltage ramps (from -120 to +60 mV) were applied every 17 s from a holding potential -40 mV. After getting stable ramp recordings in control conditions under continuous recording chamber perfusion, the drug (GABA, YM58483, or picrotoxin) dissolved in the extracellular solution was applied to the bath. After the ramp was stabilized in the presence of the drug, the latter was washed out before the next current recording. The current values were taken at -80 and -40 mV and normalized to the current in the absence of the drugs.

### Chemical compounds

**Figure 1:**
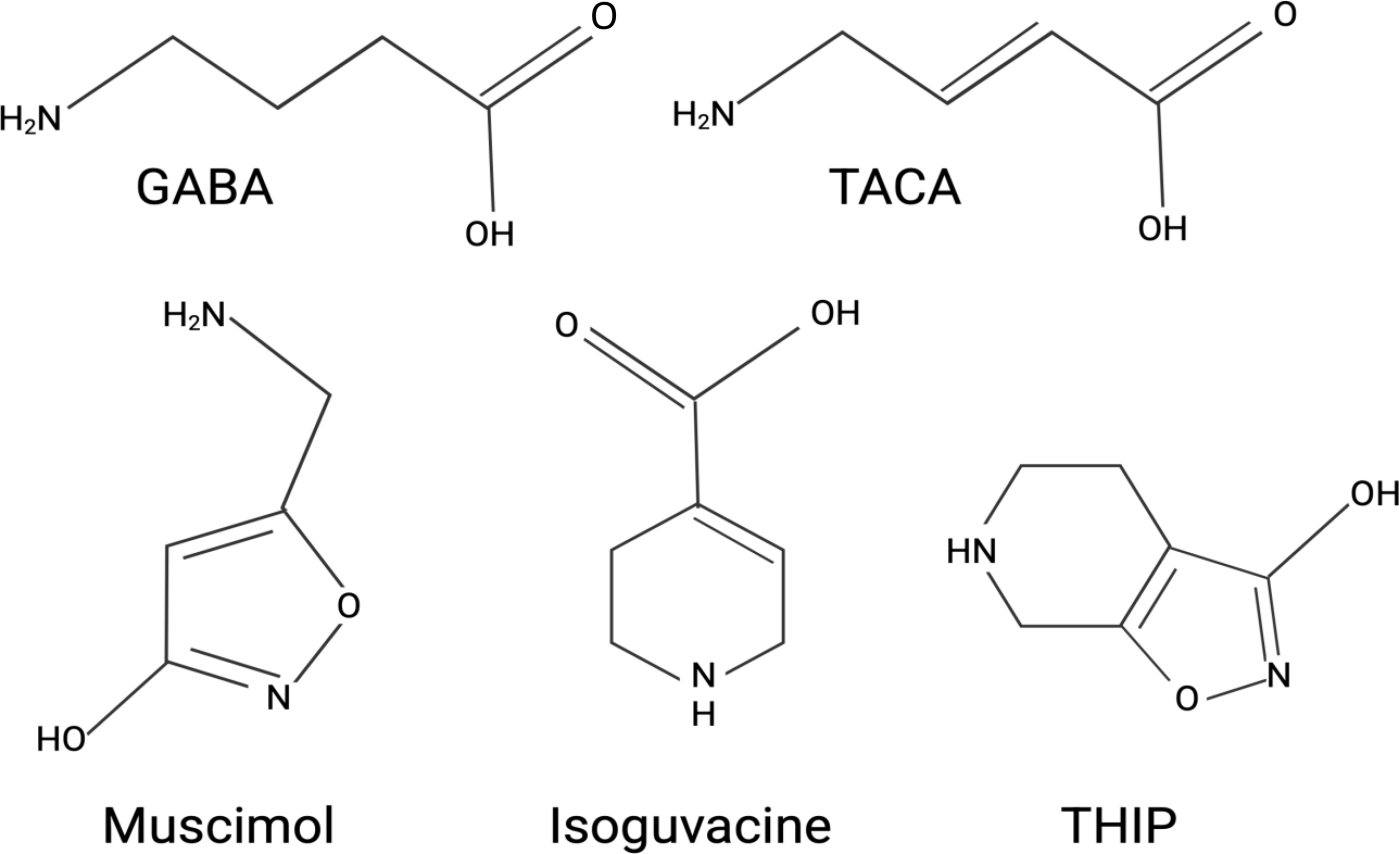
Chemical structures of GABA_A_ receptor agonists. Trans-4-aminocrotonic acid (TACA) is a GABA analog with an unsaturated carbon–carbon bond, whereas muscimol and isoguvacine are heterocyclic GABA analogs. 4,5,6,7-tetrahydroisoxazolo(5,4-c)pyridin-3-ol (THIP) is a relatively rigid analog of GABA.

The chemicals, unless otherwise specified, were purchased from Sigma-Aldrich/Merck (Steinheim, Germany). GABA_A_ receptor agonists muscimol, isoguvacine, trans-4-aminocrotonic acid (TACA), and THIP hydrochloride were purchased from Tocris (UK) and used to examine the pharmacological response of the GABA_A_ receptors (15) expressed in T cells. The agonists’ chemical structures (1, 13, 16) are shown in **Figure 1**. TACA (trans-3-aminocrotonic acid) is a GABA analog with an unsaturated carbon–carbon bond, whereas muscimol and isoguvacine are heterocyclic GABA analogs. THIP (4,5,6,7-tetrahydroisoxazolo(5,4-c)pyridin-3-ol) is a relatively rigid analog of GABA.

### Total RNA isolation and quantitative PCR

Total RNA was extracted from CD4^+^ T cells using the NucleoSpin RNA Mini kit (Macherery-Nagel, Germany) with on-column DNase treatment to remove genomic DNA. The extracted RNA was quantified with the NanoDrop 2000 spectrophotometer (Thermo Scientific, USA). Reverse transcription was performed using SuperScript reverse transcriptase IV (ThermoFisher Scientific, USA), according to the manufacturer’s protocol. Quantitative PCR was performed using qPCRBIO SYBR Mix low-ROX (PCR Biosystems, UK) and gene-specific primers (6) in a 384-well plate using a QuantStudio™ 6 Flex Real-Time PCR system (ThermoFisher Scientific, USA). The PCR cycling protocol consists of an initial denaturation step of 2 min at 95 °C, followed by 45 cycles of 95 °C for 5 s and 60 °C for 30 s. The relative expression levels of the target genes were expressed as 2^-ΔCt^ (Ct, cycle threshold) by normalizing to reference genes (*IPO8*). All samples were run in duplicate.

### Immunofluorescence staining

CD4^+^ T cells were collected, washed with PBS, and deposited on cytoslides (ThermoFisher Scientific, Sweden) using a Shandon Cytospin centrifuge (400 rpm for 4 min at room temperature (RT)). The cells were fixed with 4% paraformaldehyde (PFA) for 15 min and washed three times with PBS. Triton X-100 (0.1%) was applied for 5 min to permeabilize the cell membrane. The cells were further blocked with 5% bovine serum albumin and 10% donkey serum (Jackson ImmunoResearch) diluted in PBS for 60 min. After PBS washing, the cells were incubated with primary antibodies—rabbit anti-insulin receptor (ThermoFisher Scientific, 1:500), rabbit anti-GABRR2 (ThermoFisher Scientific, 1:600), mouse anti-GABRB3 (UC Davis, USA, 1:500)—diluted in blocking solution overnight at 4 °C, followed by incubation with a secondary antibody—AlexaFluor 488-conjugated AffiniPure Donkey Anti-Rabbit IgG (Jackson ImmunoResearch, 1:500) or Rhodamine Red X-conjugated AffiniPure Donkey anti-Mouse IgG (Jackson ImmunoResearch, 1:600)—for 1 h at RT. The cells were washed and counterstained with DAPI for 5 min. The cytoslides were mounted in ProLongTM Gold antifade reagent (ThermoFisher Scientific, USA). Images were acquired using a confocal microscope (LSM700, Zeiss) with ×63 objective.

### Data presentation

Single-cell data presentation and agonist-Ca^2+^-response data were fitted in GraphPad Prism 10 (GraphPad Software Inc., La Jolla, CA, USA). The data were fitted using nonlinear regression with a standard dose–response model:


$${\left(\frac{F}{F_{\text{median}}}\right)}_{\max}=\frac{\left[\text{agonist}\right]}{\left[\text{agonist}\right]+{\left({\text{EC}}_{50}\right)}_{\text{agonist}}}$$

where (*F*/*F*_median_)_max_ is the peak Ca^2+^ response to different concentrations of the agonist, [agonist] is the concentration of the agonist (GABA, muscimol, TACA, isoguvacine, or THIP), and (EC_50_)_agonist_ is the half-maximal concentration of the agonist.

### Statistical analysis

For the comparison of two groups, paired *t*-test or Wilcoxon test for paired data or Mann–Whitney test for unpaired data was used. The statistical significance level was set to 0.05.

## Results

### Agonist-evoked cytoplasmic Ca^2+^ response

**Figure 2:**
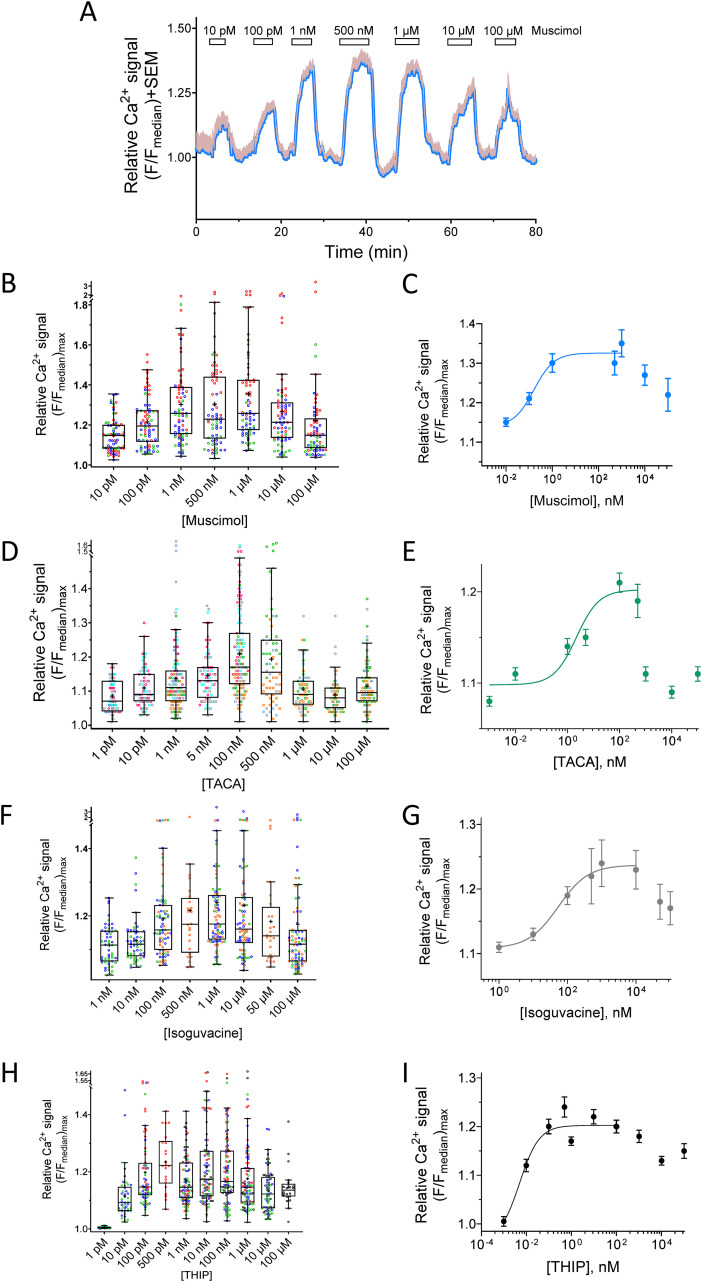
Relative calcium responses to different concentrations of the GABA_A_ receptor agonists examined in human CD4^+^ T lymphocytes. Cells were activated and cultured for 72 h, insulin (1 nM) was included for the last 24 h, and glucose concentration was 5.5 mM. **(A)** Representative mean Ca^2+^ signal intensity trace (blue line) and SEM (light pink whiskers) recorded in cells (*n* = 35 from one donor) at 72 h post-activation. The bars show the perfusion time of the drugs. Between applications of muscimol, the recording chamber was perfused with the bath solution. Relative calcium signals evoked by **(B)** muscimol (*n* = 72 cells, *N* = 6 donors), **(D)** TACA (*n* = 66 cells, *N* = 3 donors), **(F)** isoguvacine (*n* = 77 cells, *N* = 3 donors), and **(H)** THIP (*n* = 92 cells, *N* = 4 donors) in cells from different donors (marked by different colors). Mean dose–response values (mean ± SEM), from the lowest agonist concentration to the concentrations giving the maximal response for muscimol **(C)**, TACA **(E)**, isoguvacine **(G)**, or THIP **(I)** and fitted with a standard dose–response curve model.

In human-activated CD4^+^ T cells, the GABA activation of GABA_A_ receptors leads to a graded, transient increase in the intracellular Ca^2+^ signal (12). In contrast, GABA has no effect on the intracellular Ca^2+^ levels in human resting CD4^+^ T cells (**Supplementary Figure 1**). We examined if muscimol, isoguvacine, TACA, and THIP similarly induced Ca^2+^ signals in the human CD4^+^ T cells and then how they were related to the agonist concentration. Typical Ca^2+^ signal-intensity responses are exemplified by muscimol applications to the CD4^+^ T cells (**Figure 2A**). Increasing the agonist concentration evoked larger Ca^2+^ signals until a maximal response was attained, and then at supersaturating concentrations, the Ca^2+^ signal decreased in accordance with the desensitization of GABA_A_ receptors at saturating agonist concentrations (17). Muscimol is a selective and potent agonist of most GABA_A_ receptors (13). The applications of muscimol to the CD4^+^ cells revealed a dose-dependent Ca^2+^ response in the range of concentrations from 10 pM to 1 μM muscimol (**Figures 2B, C**). The relative maximum Ca^2+^ signal of approximately 1.17 was recorded at 1 μM muscimol (**Figures 2B, C**). At higher concentrations, the Ca^2+^ signal decreased. The data were fitted to a dose–response curve, and the estimated half-maximal activation concentration, EC_50_, was 0.162 nM. TACA is an agonist at heteromeric GABA_A_ receptors but is more selective for homomeric ρ receptors (13). The relative maximum Ca^2+^ signal in 500 nM TACA was 1.10 (**Figures 2D, E**). At higher concentrations, the Ca^2+^ response sharply decreased. The data were fitted to a dose–response model, and the estimated half-maximal concentration was calculated, EC_50_ = 2.2 nM. Isoguvacine is effective at many GABA_A_ receptors but has a significantly lower EC_50_ at homomeric ρ2 receptors compared with GABA (16). The relative maximum Ca^2+^ signal in 10 μM isoguvacine was 1.13 (**Figures 2F, G**). At higher concentrations, the Ca^2+^ response decreased. The data were fitted to a dose–response model, and the estimated half-maximal concentration was calculated, EC_50_ = 54.4 nM. THIP or gaboxadol is a potent GABA_A_ receptor agonist with some preference for receptors containing the δ subunit in the receptor complex (18, 19). The relative maximum Ca^2+^ signal in 100 nM THIP was 1.20 (**Figures 2H, I**). At higher concentrations, the Ca^2+^ response decreased slightly. The data were fitted to a dose–response model, and the estimated half-maximal concentration was calculated, EC_50_ = 0.005 nM.

The results show that the apparent affinity (EC_50_) and the efficacy of the induced Ca^2+^ signals varied among the agonists (**Figure 2**). The EC_50_ values are related to the potency of the compounds and resulted in the following sequence: GABA (0.005 nM) (12) ≈ THIP (0.005 nM) < muscimol (0.162 nM) < TACA (2.2 nM) < isoguvacine (54.4 nM). In contrast, efficacy, which is related to the maximal cellular Ca^2+^ signal, resulted in the following sequence: GABA (12) >> THIP > muscimol > isoguvacine > TACA. Interestingly, GABA remained both most potent and with the highest efficacy of the five agonists tested in this study.

### Expression of GABA_A_ receptor subunits in the CD4^+^ T cells

**Figure 3:**
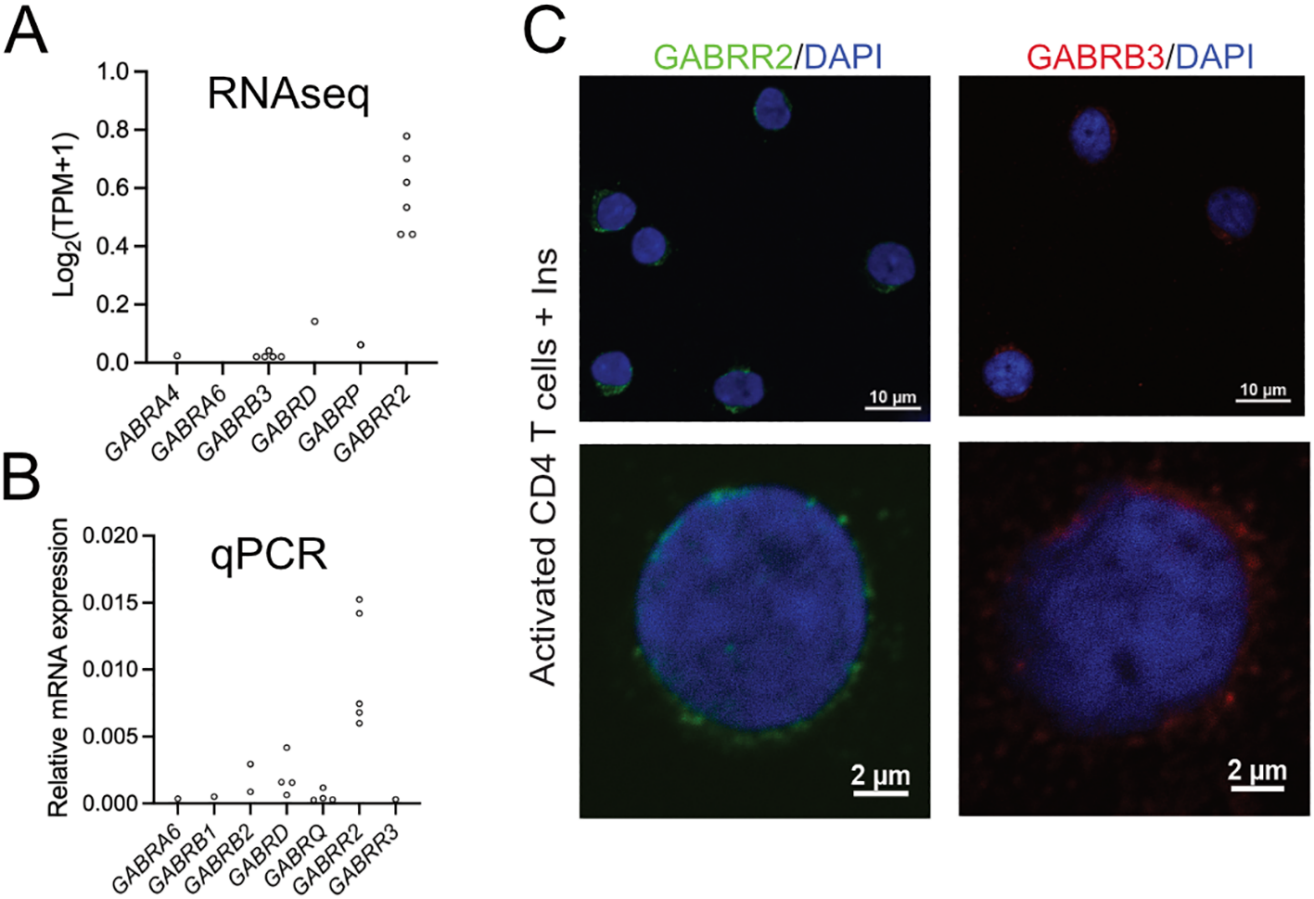
Expression of GABA_A_R subunits in activated human CD4^+^ T cells. **(A, B)** GABA_A_R subunit mRNA expression detected by RNAseq **(A)** (*N* = 6) and qPCR **(B)** (*N* = 5) in activated CD4^+^ T cells at 72 h post-activation. **(C)** Immunofluorescent staining of GABRR2 (ρ2) and GABRB3 (β3) in activated CD4^+^ T cells at 72 h post-activation. The nuclei were counterstained with DAPI. RNAseq data were extracted from the Gene Expression Omnibus (GEO) database (GSE230041).

We have previously shown that human immune cells can express a variety of GABA receptor subunits (6, 20) and that, in the CD4^+^ T cells, insulin shifts the receptors GABA EC_50_ to lower concentrations. This is, in part, related to the increased expression of the insulin receptor with time after the activation of the T cells (**Supplementary Figure 2**) as insulin enhances the expression of the GABA_A_ receptors (12). We examined the expression level of the GABA_A_ receptor subunits in the CD4^+^ T cells 72 h after activation (**Figures 3A–C**). We studied which GABA_A_ receptor subunits could be detected in the cells. Interestingly, the ρ2 subunit was most highly expressed in the activated CD4^+^ T cells (**Figures 3A–C**), but a few other subunits were expressed, albeit at low levels, including β3 that could also be detected at the protein level in the cells (**Figure 3B**). Clearly, both homomeric and heteromeric ρ-containing GABA_A_ receptors may be present in the CD4^+^ T cells.

### GABA-activated Ca^2+^ signals are reduced by CRAC and K^+^ channel blockers

**Figure 4:**
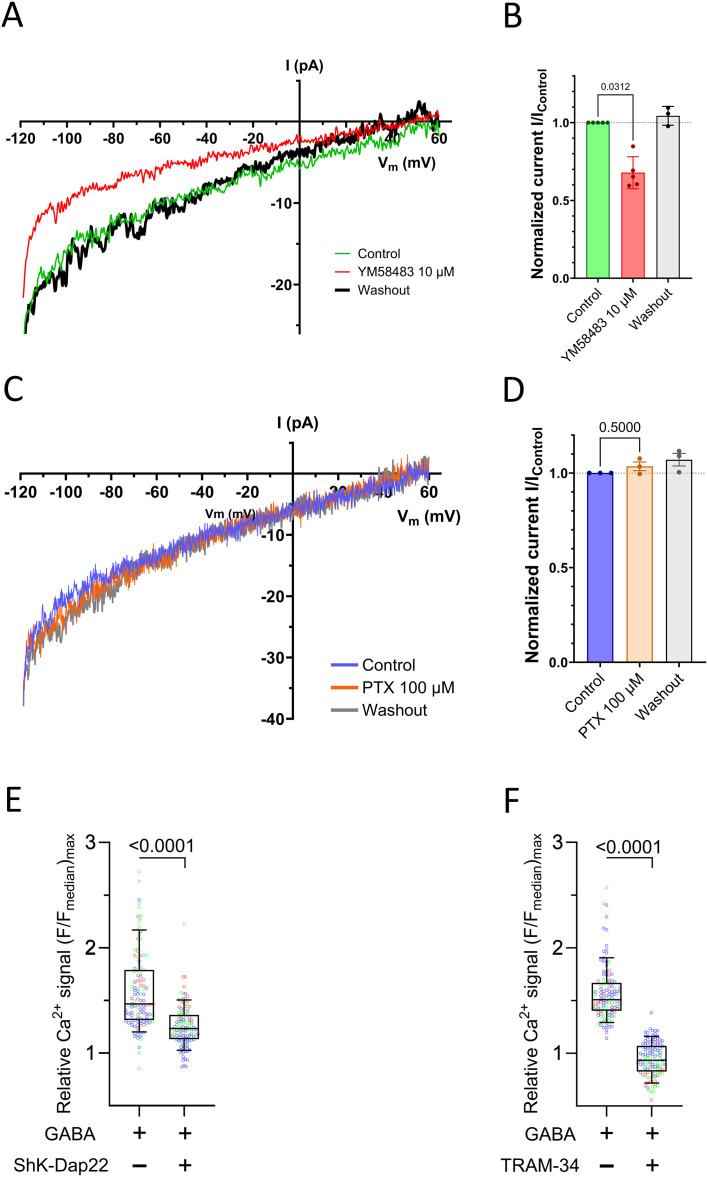
Inhibition of ion channels in human CD4^+^ T cells. Human CD4^+^ T cells derived from healthy donors were activated for 72 h in a medium with 5.5 mM glucose, and 1 nM insulin was added in the last 24 h of activation. **(A)** Inhibition of CRAC channel current by YM58483. **(B)** Statistical analysis of the YM58483 experiments (*P* = 0.0312, *n* = 5 cells from *N* = 2 donors, Wilcoxon matched-pairs signed rank test). **(C)** Picrotoxin (PTX) does not block the CRAC channel current. **(D)** Statistical analysis of the PTX experiments (*P* = 0.5, *n* = 3 cells from *N* = 2 donors, Wilcoxon matched-pairs signed rank test). (**A**–**D**) Voltage ramps of 200 ms (from -120 to +60 mV) were applied every 17 s from a holding potential of -40 mV in a whole-cell configuration. After getting stable ramp recordings in control conditions under continuous recording chamber perfusion, the drug (YM58483 or PTX) was applied to the bath. After the ramp was stabilized in the presence of the drug, the latter was washed out. The current values were taken at -80 mV at every condition and were normalized to the control current. **(E)** The GABA (500 nM)-induced increase in the intracellular Ca^2+^ levels is inhibited by ShK-Dap22 (500 pM) by 47% on average (*n* = 151 cells, *N* = 3 donors). **(F)** TRAM-34 (1 µM) inhibited the increase in the intracellular Ca^2+^ levels by almost 100% on average (*n* = 169 cells, *N* = 3 donors). The box-and-whiskers plot represents the maximum relative intracellular Ca^2+^ levels (F/F_median_)_max_ achieved during the drug application period from individual cells. The blue, green, and red dots indicate cells from a specific human donor (*p* < 0.0001).

We have previously shown the involvement of the CRAC channels in the GABA-activated cascade (12) where the acutely applied 1 μM YM58483 specifically blocked the channels (21). A higher concentration (10 μM YM58483) acutely applied also reversibly inhibited the Ca^2+^ current and the Ca^2+^-evoked signal (**Figures 4A, B**; **Supplementary Figures 3A, B**) in accordance with other studies (21–23). Picrotoxin, an inhibitor of GABA_A_ receptors (24), did not reduce the Ca^2+^ current (**Figures 4C, D**). YM58483 reduced the Ca^2+^ current by approximately 40%, indicating that even other plasma membrane Ca^2+^ channels may be activated, may be open, and may allow Ca^2+^ entry in the activated T cells. Previous studies have established that potassium ion channels not only maintain the resting membrane potential in T cells but also enhance calcium entry through the CRAC channels (25). The K_V_1.3 and/or KCa3.1 channels are expressed at a sufficiently high density so that their activation prevents the cell from depolarizing even when CRAC channels are open (25). Thus, the K^+^ channels contribute to maintaining a high driving force for Ca^2+^ influx. As the Cl^-^ equilibrium potential is more depolarized than the resting membrane potential (12, 25), it is possible that opening the GABA_A_ receptor channels will promote depolarization and increase the openings of the K_V_1.3 channel, which will enhance Ca^2+^ influx through the CRAC channels and, in turn, enhance further the activation of KCa3.1 channels (25). We examined if this coordinated channel activity took place by activating the GABA_A_ receptors in the absence or presence of blockers for these K^+^ channels, and the results are shown in **Figures 4E, F**. Both ShK-Dap22 (**Figure 4E**), a potent blocker of K_V_1.3 channels, and TRAM-34 (**Figure 4F**), a selective blocker of KCa3.1 channels, significantly reduced the GABA-activated Ca^2+^ signal in T cells. The results are consistent with the coordinated effects of the GABA_A_ receptors—K_V_1.3, KCa3.1, and CRAC channels—on the Ca^2+^ signal in T cells upon application of GABA_A_ receptor agonist to the cells.

## Discussion

**Figure 5:**
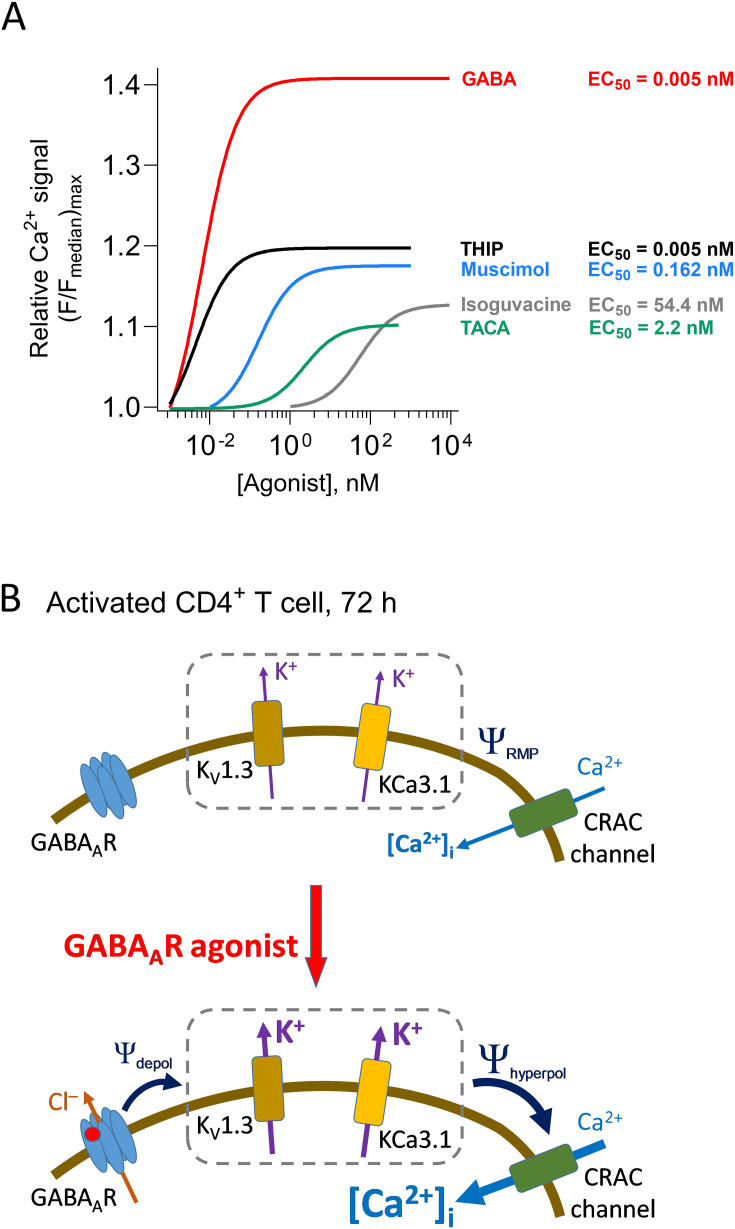
Dose–response curves of the Ca^2+^ signals for GABA_A_ receptor agonists and a model for the GABA activation of the Ca^2+^ influx in the CD4^+^ T cells. **(A)** Family of dose–response curves for the GABA_A_ receptor agonists muscimol, TACA, isoguvacine, and THIP that induced Ca^2+^ signals in the CD4^+^ T cells, activated for 72 h and incubated with insulin (1 nM) for the last 24 h (glucose concentration 5.5 mM). For comparison, the dose–response curve for GABA is also shown (12). **(B)** In activated human CD4^+^ T cells, at 72 h post-activation, the voltage-gated K^+^ channel, K_V_1.3, and the Ca^2+^-activated K^+^ channel, KCa3.1, are active and maintain the resting membrane potential (Ψ_RMP_) of the cells. When agonists of GABA_A_ receptors (GABA_A_R) are applied (red circle), the GABA_A_ receptor channels open. Since the Cl^–^ reversal potential in T cells is more positive than the resting membrane potential, opening GABA_A_ receptor channels depolarizes the cell membrane potential (Ψ_depol_) that, in turn, will result in opening many more K_V_1.3 channels that then hyperpolarize the membrane potential (Ψ_hyperpol_). This hyperpolarization of the membrane potential increases the driving force for Ca^2+^ ion influx through CRAC channels. Increased intracellular Ca^2+^ concentration then increases KCa3.1 channel activity that, together with K_V_1.3 channels, contributes to the further increase in Ca^2+^ influx through the CRAC channels.

In activated human CD4^+^ T cells, GABA binding to GABA_A_ receptors leads to a transient increase in intracellular Ca^2+^ concentrations that modulate the metabolism and secretion of inflammatory molecules from the cells (12). Here we examined the ability of several GABA_A_ receptor agonists to induce Ca^2+^ signaling in human CD4^+^ T cells. THIP, muscimol, isoguvacine, and TACA all induced graded, concentration-dependent Ca^2+^ signals in the CD4^+^ T cells, but they varied in both the apparent affinity and efficacy, and none surpassed GABA (**Figure 5A**). The GABA-activated Ca^2+^ influx was dependent on active K^+^ channels (**Figure 5B**). The effects of GABA_A_ receptor agonists on CD4^+^ T cell Ca^2+^ homeostasis enable a “tap-like” on/off effect on intracellular Ca^2+^, with significant potential to modulate the cells. Furthermore, the enabled Ca^2+^ signal may be a very effective tool when screening for new, efficacious GABA_A_ receptor agonists regulating T cell functions.

Among the plethora of potential heteromeric GABA_A_ receptors expressed in neurons and other cell types in the periphery (2, 26–28), ρ-containing receptors have a more restricted distribution and subunit composition. The ρ2 subunit can be found in the hippocampus, cerebellum, and spinal cord (29–31) but is mostly associated with the bipolar and horizontal cells in the retina (29, 32, 33). It is mostly studied as a ρ homomeric receptor in *Xenopus* oocytes (13, 16) and has also been reported to form heteromeric receptors, with at least the γ2 and potentially the α1 subunit (34–36). In human peripheral blood mononuclear cells, the ρ2 subunit is the most highly expressed GABA_A_ receptor subunit (20), similar to what we report here for CD4^+^ T cells. Furthermore, insulin enhances the expression of ρ2 after the activation of CD4^+^ T cells (12). However, as other subunits can also be expressed (11), not only homomeric but also heteromeric ρ2-containing GABA_A_ receptors may potentially be formed in CD4^+^ T cells.

How GABA_A_ receptors’ activation evokes a Ca^2+^ signal in T cells is not well understood. However, we have previously shown that the signal can be inhibited by a CRAC channel blocker at 1 μM concentration (12). The CRAC channel blocker reduced the Ca^2+^ current by 40%, indicating that even other Ca^2+^ channels may participate in the cascade. In this study, blockers of potassium channels K_V_1.3 and KCa3.1 reduced the intracellular Ca^2+^ increase. This is in line with the established, coordinated channel activation observed in T cells after T cell activation, which has been shown to regulate Ca^2+^ entry into the cells (25). Our findings demonstrate that GABA and other GABA_A_ receptor agonists can transiently activate this cascade. The Cl^–^ reversal potential is normally less negative than the resting membrane potential in human CD4^+^ T cells (12, 25). Thus, the activation of GABA_A_ receptors leads to membrane potential depolarization in the cells. In T cells, the resting membrane potential is maintained by K^+^ channels, where it depends on the state of the cells if it is predominantly regulated by K_V_1.3 or KCa3.1 (25). These channels are expressed at sufficiently high density (25) so that their activation prevents the cell from depolarizing even when the CRAC channels are open. These K^+^ channels thus maintain a high driving force for Ca^2+^ influx as long as they are open. When the GABA_A_ receptor channels are opened, the membrane potential will depolarize and a channel activation cascade starts (**Figure 5B**). K_V_1.3 channels that require depolarization for opening will open and shift the membrane potential to hyperpolarizing values, which increases Ca^2+^ influx through the CRAC channels, KCa3.1 will increase in activity with increased intracellular Ca^2+^, thus maintaining the hyperpolarized membrane potential and the inward driving force for the Ca^2+^ flux. The result of activating the channel cascade, which can be started by the GABA_A_ receptor agonists, is a transient increase in the intracellular Ca^2+^ in CD4^+^ T cells (**Figure 5B**).

We have previously shown that the GABA_A_-receptor-specific agonists (15), GABA, muscimol, and TACA, activate GABA_A_ receptor Cl^-^ currents in human CD4^+^ T cells that are blocked by picrotoxin, a specific GABA_A_ receptor open channel blocker (6, 12, 15, 37, 38). Muscimol (500 nM) or TACA (500 nM) also evoked Ca^2+^ signals that were blocked by picrotoxin (12). The GABA EC_50_ value (0.04 nM) for the GABA-activated Cl^-^ current was larger than the EC_50_ value (0.005 nM) of the GABA-evoked Ca^2+^ signal in human CD4^+^ T cells (12). This difference in the apparent affinity (EC_50_) for GABA is probably related to the high sensitivity and amplification of the Ca^2+^ signal due to the activation of the potassium channels that then increase the driving force for Ca^2+^ entry, thus enabling the sensitization of GABA signaling. The GABA apparent affinity is approximately 10^5^-fold higher in human CD4^+^ T cells than what is reported for the ρ-containing GABA_A_ receptors expressed in *Xenopus* oocytes (13) and retinal receptors (34) but similar to the extrasynaptic GABA_A_ receptors in rat CA1 pyramidal neurons (39). Whether the higher apparent affinity (lower EC_50_) in CD4^+^ T cells is related to the incorporation of other subunits than ρ2 in GABA_A_ receptors (34), different intracellular modulations or tethering (28, 40–42) or modulation by other native molecules (27, 28, 39, 41) is not known to date.

The agonists’ apparent affinity sequence for the homomeric GABA_A_ ρ1–3 receptors expressed in *Xenopus* oocytes is somewhat different from the one we reported here for the GABA-evoked Ca^2+^ signals (13, 16). The EC_50_ value for the Ca^2+^ signal evoked by GABA was shifted by approximately 10^6^-fold toward higher affinity in CD4^+^ T cells compared with the *Xenopus* oocytes’ EC_50_ for the GABA-activated Cl^-^ currents (13), whereas this difference was only approximately 10 in the CD4^+^ T cells (12). TACA, on the other hand, had lower apparent affinity and approximately 500-fold higher EC_50_ value for the Ca^2+^-evoked signal than GABA. For comparison, in heterologously expressed receptors, the TACA’s EC_50_ value based on Cl^-^ currents was approximately 10 times lower than that of GABA (13). Isoguvacine had the lowest apparent affinity of all the agonists in both systems. Interestingly, THIP, which is an antagonist at least at GABA_A_ ρ1 and ρ3 receptors (13), is, in contrast, a high-apparent-affinity agonist in human CD4^+^ T cells, similar to GABA. However, THIP has lower efficacy in inducing the Ca^2+^ response than GABA and, in this aspect, is more similar to muscimol that had intermediate EC_50_ for the Ca^2+^ response. GABA did not only have the highest apparent affinity but was also the most efficacious agonist for evoking the Ca^2+^ signal in human CD4^+^ T cells, followed by THIP and muscimol and then by isoguvacine and TACA (**Figure 5A**). At supersaturating concentrations of the agonists, the Ca^2+^ signal decreased as expected since GABA_A_ receptors desensitize faster and more completely at high agonist concentrations (17, 43). In CD4^+^ T cells, GABA not only induces transient Ca^2+^ signals in the cells but also decreases the activity and protein level of hexokinase, the gatekeeper enzyme of the glycolytic pathway (12). Whether the different agonists used in this study are similarly effective at modulating hexokinase remains to be determined. There are a number of other GABA analogues (see, e.g., (13, 16)) that may be interesting to explore and to examine whether they evoke or inhibit GABA-evoked Ca^2+^ signals in human CD4^+^ T cells. These and other compounds can potentially serve as the so-called lead compounds for drug development in drug discovery programs targeting T cell function.

## Data Availability

The original contributions presented in the study are included in the article/**Supplementary Material**. Further inquiries can be directed to the corresponding author.
