# Supplementary material for: GABAA receptor agonists modulate intracellular Ca2+ levels in activated human CD4+ T cells
Source: Front Immunol. 2026 May 8;17:1693150. doi: 10.3389/fimmu.2026.1693150 (PMC13194042; doi:10.3389/fimmu.2026.1693150)
Supplement: Supplementary file 1 [file DataSheet1.pdf]

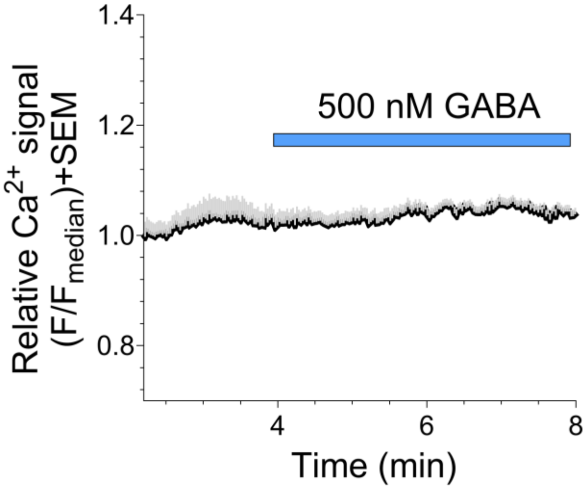

Supplementary Figure 1. Ca^{2+} signal in resting $CD4^+$ T cells. In human resting $CD4^+$ T cells GABA (500 nM) does not evoke a calcium response (n = 47 cells)

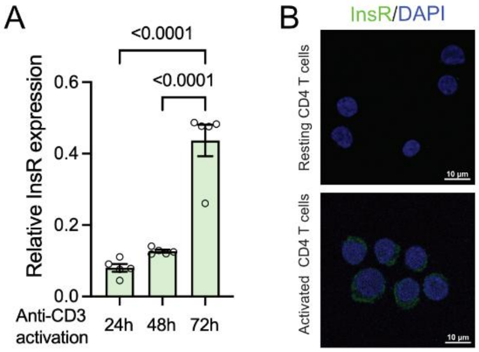

Supplementary Figure 2. Insulin receptor expression in $CD4^+$ T cells.

A. Insulin receptor (InsR) mRNA expression in $CD4^+$ T cells 24, 48 and 72 h post-activation with anti-CD3 antibody (3 µg/ml). One-way ANOVA with Dunnett's test was applied for multiple comparison (N=5). Relative expression level of *InsR* gene was expressed as $2^{-\Delta Ct}$ by normalizing to reference gene *IPO8*. **B.** Immunofluorescent staining of InsR in resting and activated $CD4^+$ T cells 72 h post-activation.

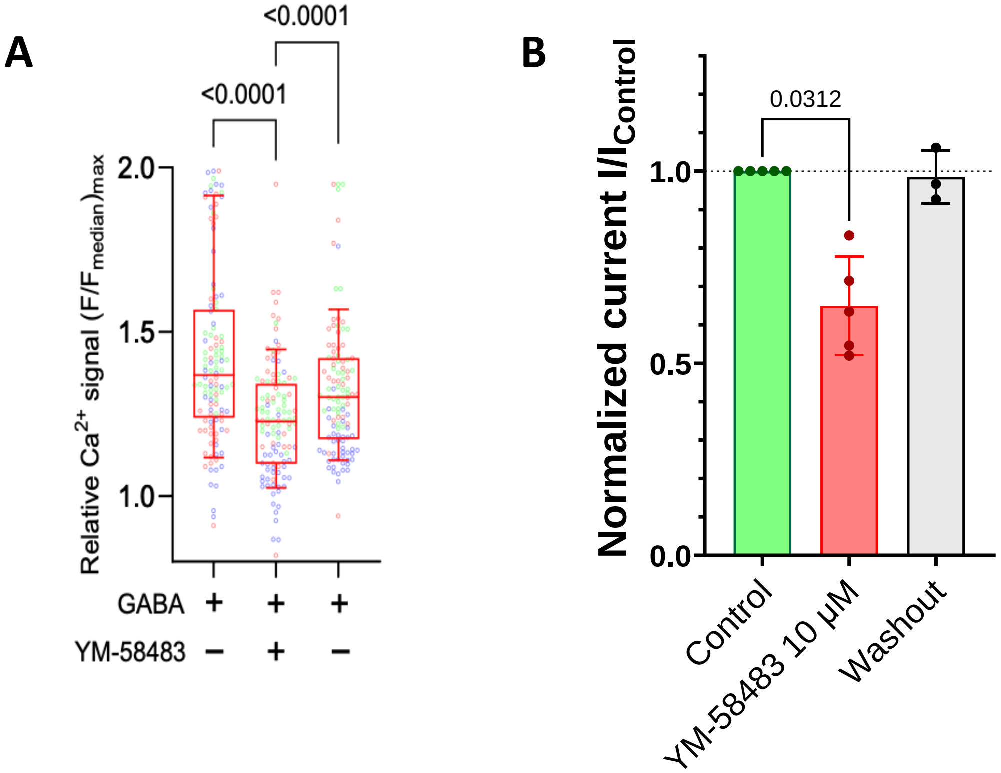

Supplementary Figure 3. Effect of CRAC channel inhibitor YM-58483 on Ca^{2+} signal and CRAC channel-mediated current. (A) GABA-mediated Ca^{2+} influx is decreased with acute application of CRAC channel inhibitor YM-58483 (10 µM). Human $CD4^{+}$ T cells derived from healthy donors were activated for 72 h in a medium with 5.5 mM glucose, and 1 nM insulin was added in the last 24 h of activation. GABA (500 nM) evoked increase in the intracellular Ca^{2+} levels is inhibited by YM-58483 (10 µM) but recovered when YM-58483 was removed (n = 116 cells, N = 3 donors). Blue, green and red dots indicate cells from a specific human donor; P < 0.0001 (nonparametric one-way ANOVA test). **(B)** 200-ms voltage ramps (from -120 to +60 mV) were applied every 17 s from a holding potential -40 mV. After getting stable ramp recordings in control conditions under continuous recording chamber perfusion, YM-58483 was applied to the bath. After the ramp was stabilized in the presence of YM-58483, the drug was washed out. The current values were taken at -40 mV and were normalized to the current in the control conditions (P = 0.0312, n = 5 cells from N = 2 donors, Wilcoxon matched-pairs signed rank test).

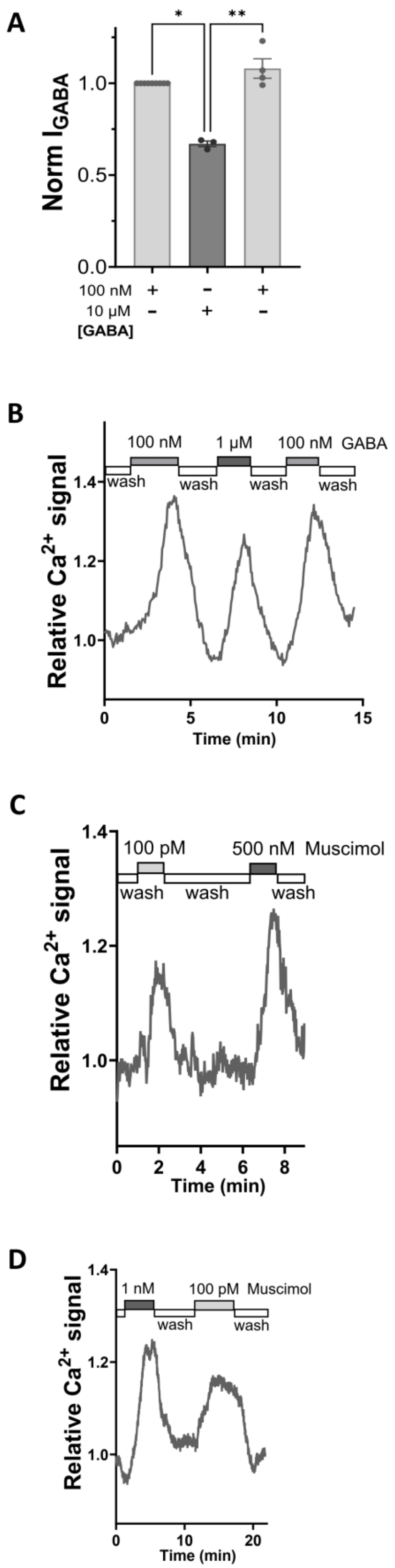
A
*
**
Norm I_{GABA}
1.0
0.5
0.0
100 nM + − +
10 µM − + −
[GABA]
B
100 nM
1 µM
100 nM
GABA
wash
wash
wash
wash
Relative Ca^{2+} signal
1.4
1.2
1.0
0
5
10
15
Time (min)
C
100 pM
500 nM
Muscimol
wash
wash
wash
Relative Ca^{2+} signal
1.4
1.2
1.0
0
2
4
6
8
Time (min)
D
1 nM
100 pM
Muscimol
wash
wash
Relative Ca^{2+} signal
1.4
1.2
1.0
0
10
20
Time (min)

Supplementary Figure 4. The dependence of GABA-induced currents and calcium signals on $GABA_A$ receptor agonist concentrations in human CD4+ T cells. (A) Application of a high GABA concentration (10 µM) after physiological GABA concentration (100 nM) decreases $GABA_A$ receptor-mediated current due to desensitization, and subsequent re-application of the initial GABA concentration (100 nM) restores the initial level of $GABA_A$ receptor-mediated current after previous GABA concentrations have been washed out. *P < 0.05, **P < 0.01, One-way ANOVA, Kruskal-Wallis test. **(B)** A recording from a T cell demonstrating the decreased calcium signal as a consequence of application of a desensitizing GABA concentration (1 µM). After washing the 1 µM GABA out of the chamber, re-application of the physiological GABA concentration (100 nM) evoked calcium response similar to the initial response. **(C)** A recording showing a concentration-response to $GABA_A$ receptor agonist muscimol. Muscimol application from a lower to a higher concentration evokes an increased calcium response in a T cell. **(D)** A recording showing a concentration-response to $GABA_A$ receptor agonist muscimol from a higher (1 nM) to a lower concentration (100 pM) leads to a decrease of the calcium response in a T cell.
